# Influence of pigment epithelium-derived factors on H_2_O_2_-induced oxidative damage and melanin synthesis in Melan-a cells

**DOI:** 10.1186/s40659-025-00657-8

**Published:** 2025-12-29

**Authors:** Yunli Cui, Fuhao Ye, Zengyao Hou, Guohao Li, Luyao Li, Hongxia Zhao, Dongfang Hu, Zhihong Yin, Lingli Chen, Hongmei Ning, Yaming Ge, Quanhai Pang

**Affiliations:** 1 College of Veterinary Medicine, Shanxi Agricultural University, Taigu, 030801 Shanxi China; 2 College of Animal Science and Veterinary Medicine, Henan Institute of Science and Technology, Xinxiang, , 453003 Henan China

**Keywords:** Melanocytes, Oxidative stress, Apoptosis, Melanin synthesis

## Abstract

**Background:**

Previous studies have demonstrated that oxidative stress and melanogenesis are regulated by the Wnt/β-catenin signaling pathway. However, the precise mechanism by which the PEDF/Wnt/β-catenin axis modulates apoptosis and melanogenesis remains unclear.

**Methods:**

Cell viability and mortality rates were assessed using CCK-8 assays and lactate dehydrogenase (LDH) release assays. Mitochondrial ultrastructural changes were analyzed by transmission electron microscopy (TEM). Changes in the mitochondrial membrane potential (ΔΨm) were assessed using the JC-1 fluorescent probe. The effects of PEDF on protein and gene expression were evaluated by Western blotting and quantitative real-time polymerase chain reaction (qRT-PCR).

**Results:**

As the concentration of H_2_O_2_ increased, the cell survival rate decreased, which activated the Wnt/β-catenin signaling pathway and increased the apoptosis rate and melanin production. PEDF reversed the H_2_O_2_-induced decrease in cell viability and increase in mortality rate in Melan-a cells while ameliorating the impairment of the mitochondrial membrane potential. PEDF ameliorated H_2_O_2_-induced protein damage and lipid peroxidation and reduced apoptosis in Melan-a cells. PEDF treatment significantly decreased the protein expression levels of β-catenin, Wnt3a, and Dvl2 (*P* < 0.05) and reduced the protein levels of Bax and Caspase-3 (*P* < 0.01) in H_2_O_2_- and BML-284-treated Melan-a cells. Furthermore, PEDF significantly reduced the H_2_O_2_- and BML-284-induced increases in the MITF and TYR protein levels (*P* < 0.01).

**Conclusions:**

These results suggest that PEDF can reduce H_2_O_2_-induced oxidative damage and melanin production in Melan-a cells by inhibiting the activation of Wnt/β-catenin signaling pathway activation. These findings provide a theoretical basis for human oxidative stress and pigment deposition-related diseases.

**Supplementary Information:**

The online version contains supplementary material available at 10.1186/s40659-025-00657-8.

## Background

Oxidative stress is a pathological state characterized by the excessive accumulation of reactive oxygen species (ROS) and reactive nitrogen species (RNS) in the body, which overwhelms the endogenous antioxidant defense capacity. By inducing oxidative damage to biological macromolecules such as DNA, proteins, and lipids, oxidative stress directly contributes to cellular dysfunction and tissue injury [1], emerging as a core pathological mechanism underlying cardiovascular diseases [2], diabetes mellitus [3], cancer [4, 5], and neurodegenerative disorders [6]. In cutaneous melanocytes, the primary sources of ROS include ultraviolet radiation, melanin synthesis, and activation of NADPH oxidases (NOX). Excessive ROS can induce oxidative DNA damage, aberrant activation of signaling pathways, and epigenetic modifications, thereby driving the initiation and progression of vitiligo and melanoma [7–10].

The Wnt/β-catenin signaling pathway plays a central role in embryonic development, cell proliferation, and tissue homeostasis [11, 12], while its aberrant activation can promote inflammatory [12, 13], neurological disorders [14, 15], metabolic disorders [16], tissue fibrosis [17], and cancer [18] progression. Recent studies have identified pigment epithelium-derived factor (PEDF) as an endogenous inhibitor of this pathway, exerting critical regulatory functions in tissues with high expression such as melanocytes [19, 20]. PEDF not only suppresses tumor proliferation and metastasis by antagonizing Wnt/β-catenin signaling [21–25], but also exhibits significant antioxidant stress activity, including inhibiting ROS generation, alleviating oxidative damage-induced apoptosis, and protecting tissues from pathological insults such as hyperglycemia [26–28].

Melanin formation is a complex biological process regulated by multiple factors, which primarily occurs within melanosomes (lysosome-related organelles) and involves the precise coordination of synthesis, storage, and transport. Microphthalmia-associated transcription factor (MITF), a key regulator of melanocyte differentiation and function belonging to the bHLH-Zip family [29], drives melanin synthesis by directly regulating the expression of target genes such as tyrosinase (TYR), tyrosinase-related protein 1 (TRP-1), and dopachrome tautomerase (DCT) [30, 31]. The Wnt/β-catenin signaling pathway promotes β-catenin accumulation by inhibiting GSK-3β activity, thereby activating MITF [32], studies have confirmed that Wnt3a can induce melanocyte differentiation and pigment deposition through this pathway [33]. Furthermore, pigment epithelium-derived factor (PEDF), as an upstream regulatory molecule of this pathway, is involved in melanogenesis, although its specific mechanism remains to be further elucidated.

Therefore, to investigate whether PEDF protects Melan-a cells from H_2_O_2_-induced oxidative damage and inhibits melanin production by blocking the Wnt/β-catenin signaling pathway, we treated Melan-a cells with H_2_O_2_ and then observed the effects of PEDF and BML-284 (a Wnt/β-catenin pathway agonist) on oxidative damage and melanogenesis.

## Materials and methods

### Cell culture and treatment

Melan-a cells (murine melanocytes) were provided by Xinxiang Medical University. Melan-a cells were cultured in RPMI 1640 medium supplemented with 10% fetal bovine serum (FBS; Sijiqing, Zhejiang, China), 100 U/mL penicillin, and 100 µg/mL streptomycin (Solarbio, Beijing, China), with subculturing performed every 2 days. The cells were cultured at 37 °C in a humidified atmosphere containing 5% CO_2_ and 95% air. Upon reaching appropriate confluence, cells were seeded into 6-well plates (8 × 10^5^ cells/well in 2 mL) or 96-well plates (1 × 10^5^ cells/well in 100 µL) and cultured for an additional 24 h before treatment with H_2_O_2_ and/or PEDF(Cells were cotreated with 0.8 mM H_2_O_2_ and 100 ng/mL PEDF simultaneously for 12 h). After 12 h, cell morphology, cell viability, and cell mortality were observed, or cells from each group were collected for subsequent experiments.

BML-284 (a Wnt signaling activator, CAS No.: 853220-52-7), was dissolved in DMSO to prepare a 10 mM stock solution and stored at -80 °C. For experiments, Melan-a cells were pretreated with 10 µM BML-284 (final DMSO concentration ≤ 0.1%) for 2 h. After pretreatment, the medium containing BML-284 was removed, and the cells were then co-treated with H_2_O_2_ and/or PEDF for 12 h.

### Morphological observation and cell viability assay

After treatment with different concentrations of H_2_O_2_ for 12 h, cell morphology was observed using an inverted microscope (Nikon, Japan). The cells were washed twice with fresh culture medium, after which 100 µL of fresh medium and 10 µL of Cell Counting Kit-8 (Solarbio, CA1210, China) solution were added to each well. After incubation in a cell culture incubator for 2 h, the absorbance was measured at 450 nm using a multifunctional microplate reader (MultiScan Spectrum, Thermo Fisher, Waltham, Massachusetts), and the cell survival rate was calculated.

### Cell death rate by lactate dehydrogenase assay

Cell mortality was evaluated using the Lactate Dehydrogenase Cytotoxicity Assay Kit (Nanjing Jiancheng Bioengineering Institute, China) following the manufacturer’s instructions. Briefly, 20 µL of cell culture supernatant, 5 µL of double-distilled water, 25 µL of substrate buffer, and 2 µL of reduced nicotinamide adenine dinucleotide (NADH) were mixed in a 96-well plate. After incubation at 37 °C for 15 min, 25 µL of 0.4 mol/L 2,4-dinitrophenylhydrazine was added, and the mixture was further incubated at 37 °C for 15 min. Finally, 250 µL of 0.2 µmol/mL sodium pyruvate standard solution was added, mixed thoroughly, and the plate was allowed to stand at room temperature for 5 min. Absorbance was measured at 450 nm using a microplate reader, and LDH activity was calculated accordingly.

### Wound healing assay

A 10 µL sterile pipette tip (ensuring no tip damage) was used to create scratches. Prior to scratching, horizontal positioning lines with an interval of 0.5–1 cm were pre-drawn on the back of the 6-well plate using a ruler and marker pen. The tip was held perpendicular to the bottom of the well and the cell monolayer at a constant speed along the positioning lines. For the same batch of experiments, a single pipette tip was used to perform all scratches with uniform force in a single pass, avoiding repeated scraping. Immediately after scratching, cells were gently rinsed three times with PBS to remove detached cells. Images of cell migration were captured under a 10× microscope at 0 and 24 h, respectively. Images were analyzed using ImageJ software, with initial scratch widths measured 10 times per group (mean value calculated) and normalized to the scratch area at 0 h to ensure the coefficient of variation of scratch width within groups was < 10%. Furthermore, pre-experiment validation showed that performing scratches when cell confluence reached 100% with uniform cell status could further reduce width deviation [34].

### Cell proliferation

EdU was diluted with complete medium to a final concentration of 10 µM. The original medium was discarded, and 100 µL of EdU-containing medium was added to each well, followed by incubation for 2 h. After the culture medium was discarded, the cells were fixed with 50 µL of 4% paraformaldehyde for 30 min at room temperature. Permeabilization was performed using 0.5% Triton X-100 for 10 min. The permeabilized cells were then incubated with 100 µL of 1× Apollo staining solution on a decolorizing shaker for 30 min, followed by 2–3 washes (10 min each). After the permeabilization agent was discarded, the cells were incubated with 1× Hoechst 33,342 (100 µL/well) for 30 min on a decolorizing shaker to stain DNA staining. The cells were then washed three times with PBS and observed under an inverted fluorescence microscope.

### Determination of reactive oxygen species generation

Intracellular ROS generation was measured using the fluorescent probe 2′,7′-dichlorodihydrofluorescein diacetate (H2DCFDA) (Molecular Probes) from Beyotime Biotechnology. After cotreatment with 0.8 mM H_2_O_2_ and 100 ng of PEDF for 12 h, the cells were incubated with 10 µM DCFH-DA for 30 min at 37 °C and then imaged using an inverted fluorescence microscope (Nikon, Japan). Furthermore, the cells were washed, centrifuged, and resuspended in PBS, and the fluorescence intensity was measured using a fluorescence microplate reader (Ex = 490 nm, Em = 516 nm).

### Measurement of SOD and MDA activities

After cell treatment, the SOD activity of each group was determined using a total SOD activity assay kit (S0101S, Beyotime, China). Cells were washed twice with pre-cooled PBS, lysed with RIPA buffer, and protein concentration was quantified by the BCA method. Protein samples were adjusted to 1 µg/µL. In a 96-well plate, 20 µL of sample, 1 µL of enzyme working solution, 8 µL of WST-8 chromogenic reagent, and 151 µL of reaction buffer (pH 7.4 PBS) were mixed. After incubation at 37 °C for 30 min in the dark, absorbance was immediately measured at 450 nm using a microplate reader, and SOD activity was calculated according to the standard curve.

Cell sample preparation was performed as described for the SOD assay. Briefly, 100 µL of supernatant was mixed with 200 µL of TBA working solution (containing 0.37% thiobarbituric acid, 15% trichloroacetic acid, and 0.25 M hydrochloric acid). The mixture was incubated in a boiling water bath for 15 min, cooled to room temperature, and centrifuged at 1000×*g* for 10 min to remove precipitates. A 200 µL aliquot of the supernatant was collected, and absorbance was measured at 532 nm. The MDA content was calculated according to the standard curve.

### Mitochondrial membrane potential analysis

After cell treatment, the medium was discarded, and cells were gently washed once with pre-warmed PBS. Subsequently, the cells were processed in accordance with the instructions of a mitochondrial membrane potential assay kit (JC-1, Jiangsu KeyGEN BioTECH Co., Ltd., China). 1 mL of JC-1 working solution (10 µg/mL, diluted in staining buffer) was added, and the cells were incubated at 37 °C for 20 min in the dark. The supernatant was aspirated, and the cells were washed twice with 1× Incubation Buffer. After adding 1 mL of cell culture medium, cells were observed and imaged under a fluorescence microscope (Nikon, Japan) to evaluate fluorescence intensity.

### Transmission electron microscopy

After cotreatment of cells with 0.8 mM H_2_O_2_ and 100 ng of PEDF for 12 h, the culture medium was discarded. The cells were washed three times with precooled PBS, collected in 1.5 mL centrifuge tubes, and centrifuged at 1000 rpm for 10 min. After removing the PBS, the cells were fixed with 2.5% glutaraldehyde for 2 h at room temperature. For electron microscopy, 70–90 nm thick sections were collected on copper grids and stained with 5% uranyl acetate and Reynolds’ lead citrate. Images were acquired using a JEOL JEM-1230 transmission electron microscope (JEOL USA, Inc., Peabody, Massachusetts) equipped with a Gatan Ultrascan 4000 digital camera (Gatan Inc., Pleasanton, California) [35].

### Apoptosis detection

After cotreatment H_2_O_2_ and PEDF for 12 h, the cells were digested with EDTA-free trypsin. Following termination of digestion, cells were collected by centrifugation at 300×*g* for 5 min (adjusted to 1 × 10^6^ cells/mL). Subsequently, 400 µL of 1× Binding Buffer, 5 µL of Annexin V-PE (protected from light), and 5 µL of Propidium Iodide were added sequentially, gently mixed, and incubated at room temperature for 15 min in the dark. Samples were analyzed by flow cytometry within 1 h of staining. To morphologically verify the occurrence of apoptotic events, cell nucleus were stained with DAPI (Solarbio, Beijing, China) stock solution at room temperature for 5 min. Cells were washed three times with pre-cooled PBS (5 min per wash) and nuclear morphology was analyzed using a fluorescence microscope. The experiments were performed in triplicate.

### Determination of melanin content and tyrosinase activity

The melanin content in melanocytes was determined following previously described methods. Briefly, Melan-a cells were treated with H_2_O_2_, PEDF, and BML-284 for the indicated time periods, then harvested (1 × 10^6^ cells per sample). For melanin content measurement, the cells were incubated in 1 M NaOH at 80 °C for 1 h, and the absorbance was measured at 475 nm with three replicates per group. For tyrosinase activity assay, the collected cells were incubated with 2 mM L-DOPA (Sigma-Aldrich, Shanghai, China) at 37 °C for 1 h, and the absorbance was detected at 475 nm using a microplate reader with three replicates per group. Both melanin content and tyrosinase activity were normalized to the values of the control group.

### Total RNA isolation, qRT-PCR

Total RNA was isolated from treated cells using TRIpure Reagent (RP1001, Bioteke, Beijing, China) according to the manufacturer’s recommendations. The concentration of total RNA was measured using a NanoDrop 2000 spectrophotometer (Thermo Fisher Scientific, USA), and its quality was evaluated by determining the OD260/280 ratio and performing 1% agarose gel electrophoresis. For each RNA sample, 1 µg of total RNA was reverse-transcribed into cDNA using M-MLV Reverse Transcriptase (M170A, Promega) and random primers in Professional Thermocycler (Biometra, Germany). Quantitative real-time PCR was performed in triplicate with each cDNA sample by using the primers for Caspase-3, Bax, Bcl-2, Wnt3a, β-catenin, Dvl2 and SYBR^®^ Green PCR Kit (Qiagen, Germany) by QuantStudio 5 Real-Time PCR Instrument (Thermo Fisher Scientific, USA) under the following: initial denaturing at 95 °C for 30 s, 40 cycles at 95 °C for 5 s, annealing at 60 °C for 20 s, and 72 °C for 20 s. The experiments were performed in triplicate. Data was analyzed using the 2^−ΔΔCt^ method and the transcriptional level of each gene was normalized to housekeeping gene β-actin [36, 37]. Table 1 lists the sequences of primers used in this study.

**Table 1:** Primer sequences for qRT-PCR

Gene name	Primer sequences (5′–3′)	Orientation
β-actin	TTGCTGACAGGATGCAGAAG	Forward
	ACATCTGCTGGAAGGTGGAC	Reverse
Caspase-3	GGAGCAGCTTTGTGTGTGTG	Forward
	TCCAGGAATAGTAACCAGGTGC	Reverse
Bax	GCAGGGAGGATGGCTGGGGAGA	Forward
	TCCAGACAAGCAGCCGCTCACG	Reverse
Bcl-2	CGACTTTGCAGAGATGTCCA	Forward
	CATCCACAGAGCGATGTTGT	Reverse
Wnt3a	AGGACCCATCTGATTCCCCA	Forward
	CTTGTGGCAGATGGGCTGTA	Reverse
β-catenin	GCTGCTGTCCTATTCCGAATGTCTG	Forward
	GGCACCAATGTCCAGTCCAAGATC	Reverse
Dvl2	CACCGAAGGCGAAGGAAGCAG	Forward
	TGAGCGTGACCGTGATGATGTTG	Reverse

### Western blotting analysis

After treatment with H_2_O_2_ and PEDF for 12 h, cells in each group were lysed with pre-cooled RIPA lysis buffer (R0010, Solarbio, China) containing protease and phosphatase inhibitors (AR1182-1, Solarbio, China). Proteins (20 µg/sample) were separated by polyacrylamide gel electrophoresis (PAGE) and transferred onto polyvinylidene fluoride (PVDF) membranes at 100 V for 40 min. The membranes were blocked with 5% skim milk for 2 h and subsequently incubated with primary antibodies at 4 °C overnight. After incubation with the secondary antibody (1:5000, ZSGB-BIO, #ZB-2301) at room temperature for 2 h, the membranes were incubated with enhanced ECL ( Wenyuange, Shanghai, China) solution to visualize the protein bands. The experiments were performed in triplicate. Densitometric analysis of immunoblot bands was performed using Image J software (https://imagej.nih.gov/ij/), and protein levels were normalized to β-actin. Statistical analysis was conducted using GraphPad Prism 9.5. Table 2 lists the antibodies used in this study.

**Table 2:** Antibodies used in the experiment

Antibody	Concentration	Observed MW (kDa)	Source
β-actin	1:3000	42	Goat anti-rabbit IgG
Caspase-3	1:1500	17	Goat anti-rabbit IgG
Bax	1:1000	21	Goat anti-rabbit IgG
Bcl-2	1:1000	26	Goat anti-rabbit IgG
Wnt3a	1:800	42	Goat anti-rabbit IgG
β-catenin	1:6000	92	Goat anti-rabbit IgG
Dvl2	1:4000	90–95	Goat anti-rabbit IgG
MITF	1:2000	59–65	Goat anti-rabbit IgG
TYR	1:2500	70	Goat anti-rabbit IgG

### Statistical analysis

All data were analyzed using GraphPad Prism 9.5, with statistical analysis performed via one-way analysis of variance (ANOVA) followed by Tukey’s post-hoc multiple comparison test. All key experiments in this study were independently repeated three times, with each repetition including biological and technical replicates. Each biological replicate was conducted with three technical replicates, and data are presented as the mean ± SD from three independent experiments. *P* < 0.05 was considered statistically significant, and *P* < 0.01 was deemed highly significant.

## Results

### Effects of H_2_O_2_ on Melan-a cell viability and cellular functions

**Fig. 1:**
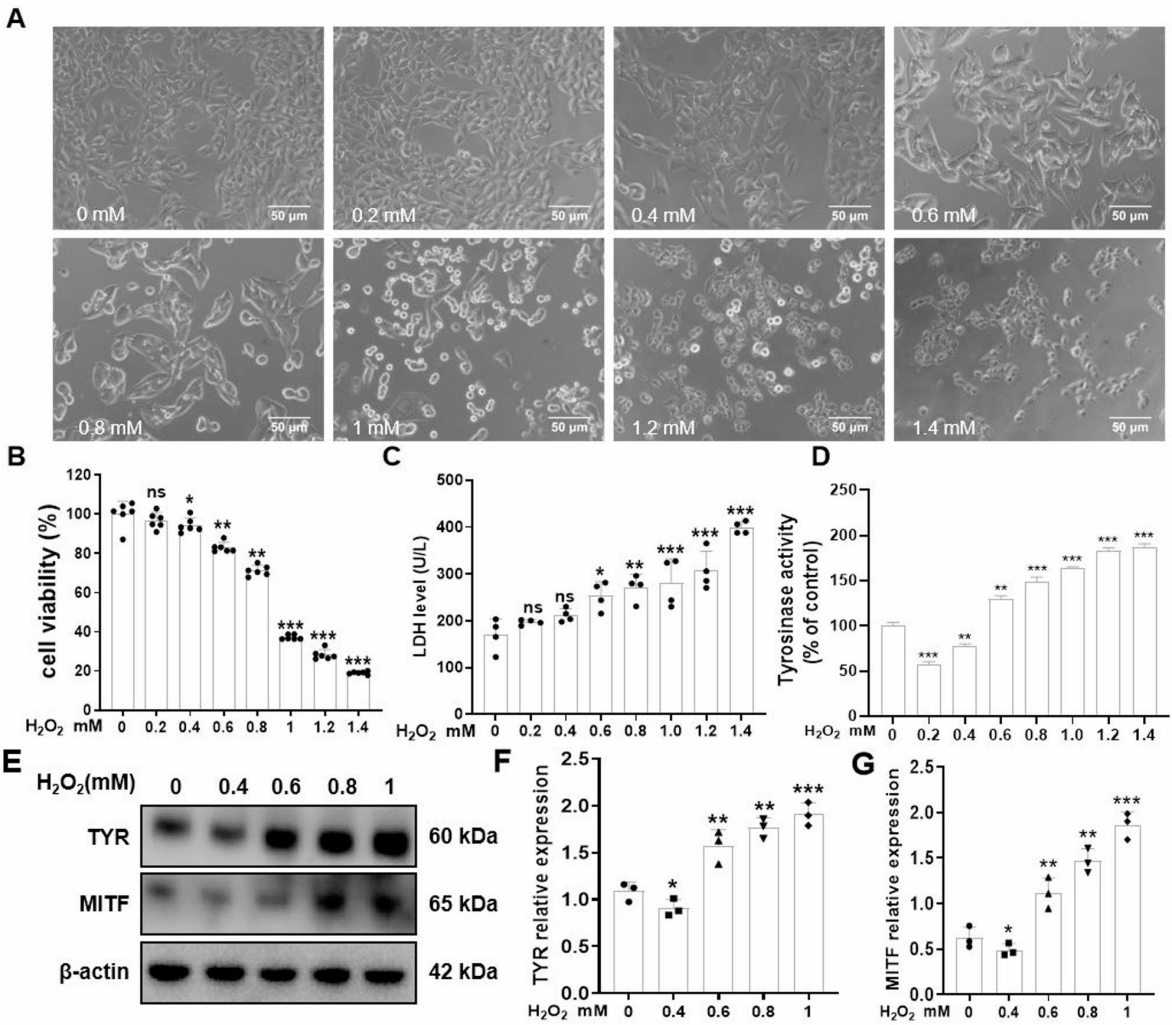
Effects of H_2_O_2_ on morphology, cytotoxicity, and melanogenesis in Melan-a Cells. Melan-a cells were treated with H_2_O_2_ at concentrations of 0, 0.2, 0.4, 0.6, 0.8, 1.0, 1.2, and 1.4 mM for 12 h, respectively. **A** Cell morphology was observed and photographed using an inverted microscope. Scale bar = 50 μm. **B** Cell viability was determined using the CCK-8 assay. **C** The LDH in the cell supernatant. **D** Results of TYR activity assay. **E** The protein expression levels of TYR and MITF in Melan-a cells after treatment with different concentrations of H_2_O_2_ for 12 h were detected by Western blotting analysis. Representative blots are shown, and β-actin served as a loading control. **F**, **G** Densitometric analysis was performed on the representative electrophoretograms for quantitative assessment. The data are presented as the mean ± SD of three independent experiments. ^*^P < 0.05 and ^**^P < 0.01, compared with the control group

After Melan-a cells were treated with different concentrations of H_2_O_2_ for 12 h, the results showed that: no morphological changes were observed in Melan-a cells treated with 0–0.4 mM H_2_O_2_, whereas cells treated with 0.6–1.4 mM H_2_O_2_ exhibited shrinkage, decreased viability, and a dose-dependent increase in the mortality rate (*P* < 0.01; Fig. 1A, B). Compared with the control, 0.8 mM H_2_O_2_ significantly reduced cell viability and increased LDH release (*P* < 0.01; Fig. 1C ). To assess the impact of H_2_O_2_ on melanogenesis in Melan-a cells, we quantified TYR activity and key melanin production markers. Intriguingly, low concentrations of H_2_O_2_ suppressed melanin synthesis, as evidenced by reduced TYR activity and downregulated protein expression of MITF and TYR. In contrast, high concentrations of H_2_O_2_ paradoxically increased melanogenic activity, with elevated TYR activity and upregulated MITF and TYR expression (*P* < 0.01; Fig. 1D–G).

### Effects of H_2_O_2_ on the Wnt/β-catenin signaling pathway and apoptosis-related proteins 

**Fig. 2:**
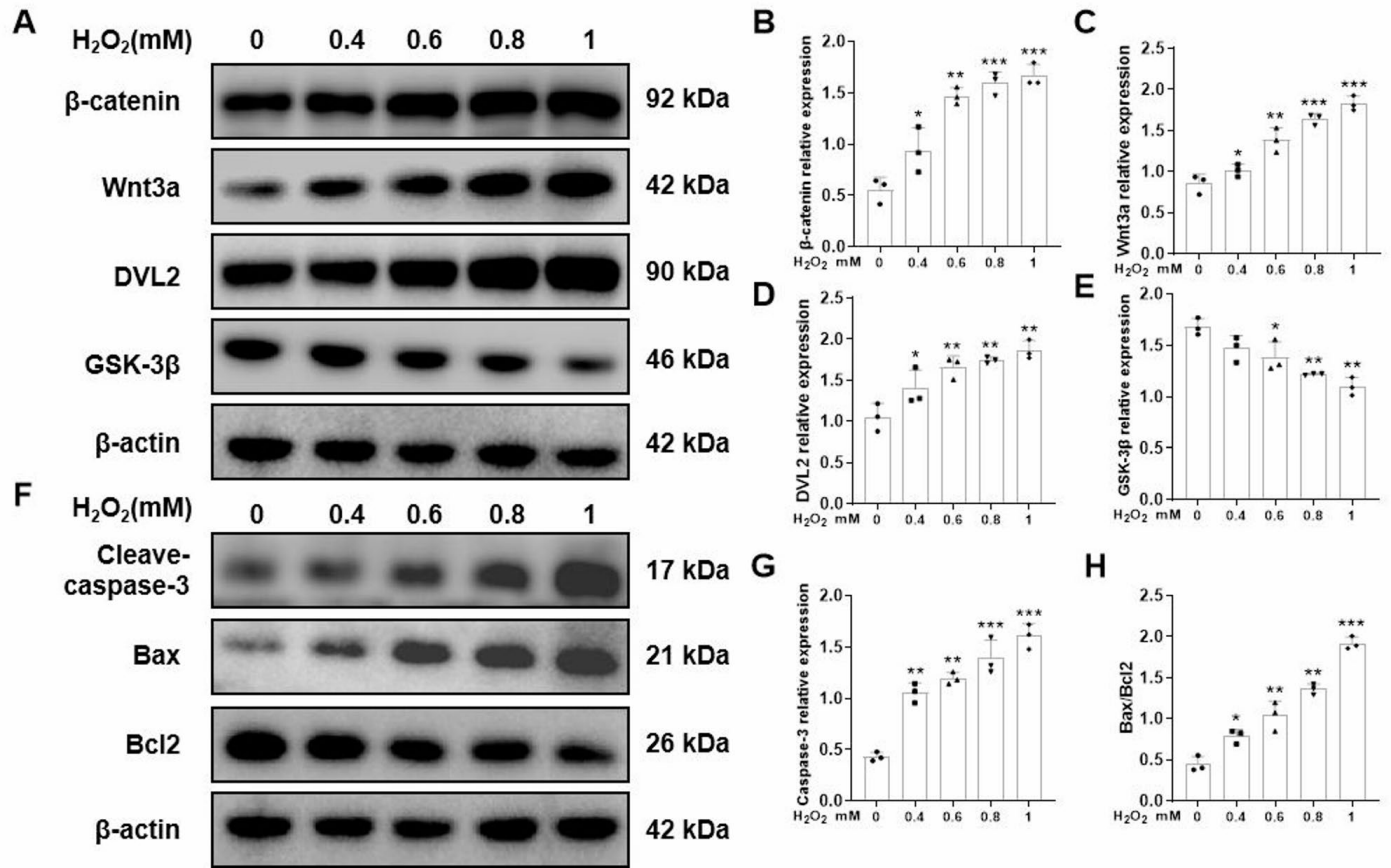
Effects of H_2_O_2_ on the Wnt/β-catenin signaling pathway and apoptosis-related proteins. Melan-a cells were treated with 0, 0.4, 0.6, 0.8, and 1.0 mM H_2_O_2_ for 12 h. The expression levels of β-catenin, Wnt3a, Dvl2, GSK3β (**A**), and apoptosis-related proteins Caspase-3, Bax, and Bcl-2 (**F**) are shown. Representative electrophoretograms are presented, with β-actin used as a loading control. **B**–**E**, **G** and **H** Densitometric analysis was performed on the representative electrophoretograms for quantitative assessment. The data are presented as the mean ± SD (*n* = 3). ^*^P < 0.05, ^**^P < 0.01, and ^***^P < 0.001, compared with the control group

After Melan-a cells were treated with different concentrations of H_2_O_2_, the Wnt/β-catenin signaling pathway was activated, as evidenced by the upregulated expression of related proteins, such as Wnt3a, β-catenin, and Dvl2, along with the downregulated expression of GSK-3β, all in a dose-dependent manner (*P* < 0.01; Fig. 2A–E). The expression levels of the proapoptotic proteins Caspase-3 and Bax were significantly increased, whereas those of the antiapoptotic protein Bcl-2 were markedly decreased, all in a dose-dependent manner (*P* < 0.01; Fig. 2F–H).

### Optimization of PEDF concentration and its protective effects against H_2_O_2_-induced damage in Melan-a cells

**Fig. 3:**
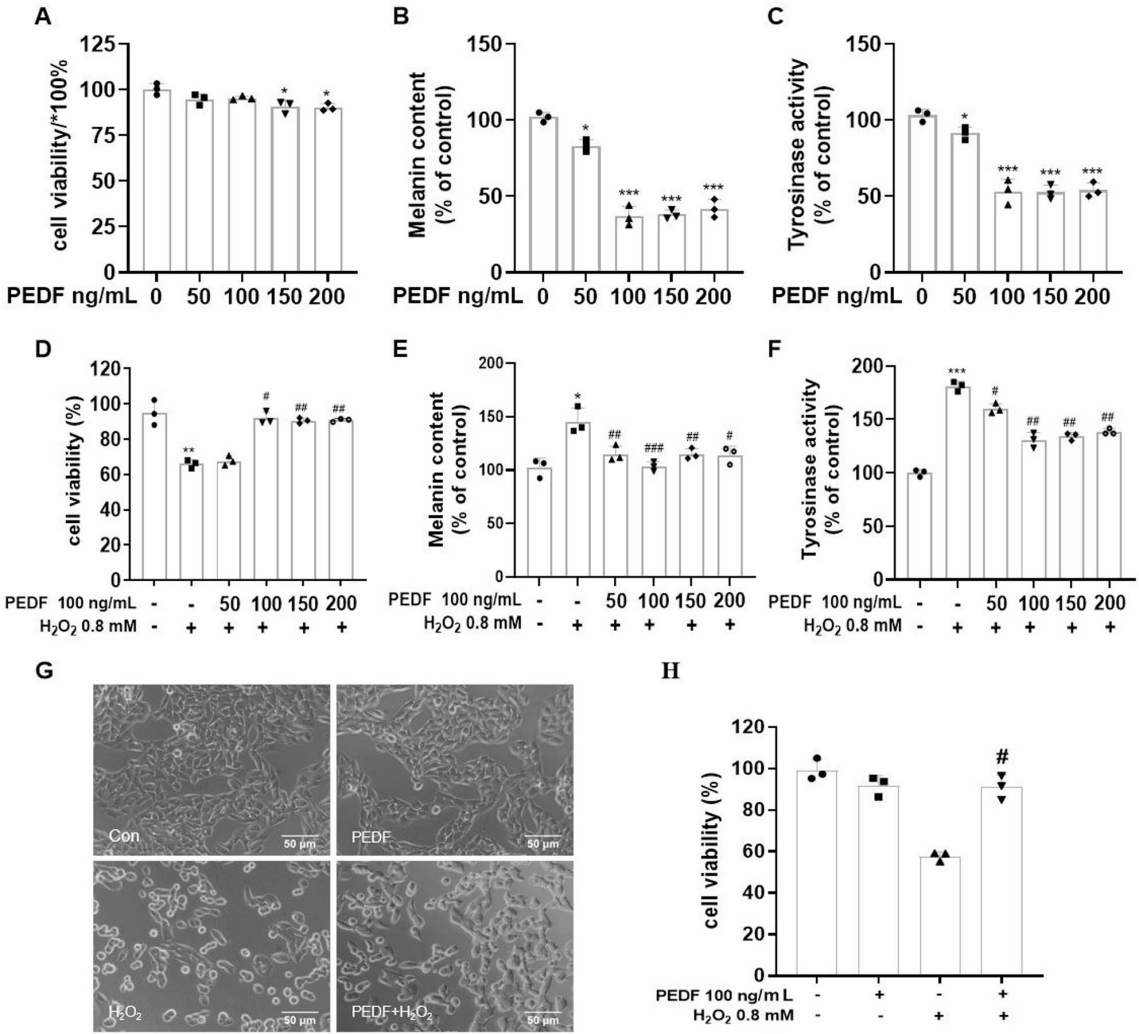
Dual protective and anti-pigmentary effects of PEDF in H_2_O_2_-damaged melanocytes. Melan-a cells were treated with different concentrations of PEDF (0–200 ng/mL) alone or in combination with 0.8 mM H_2_O_2_ for 12 h. Cell viability was determined using the CCK-8 assay (**A**, **D**, **H**). Melanin content was measured by alkaline lysis method (**B**, **E**), tyrosinase activity was detected via spectrophotometry (L-DOPA oxidation method) (**C**, **F**), and cell morphological changes were observed (**G**). Scale bar = 50 μm. The data are presented as the mean ± SD (*n* = 3). ^*^P < 0.05, ^**^P < 0.01, and ^***^P < 0.001, compared with the control group; ^#^*P* < 0.05 and ^##^*P* < 0.01, compared with the H_2_O_2_ group

To investigate the protective effect of PEDF on Melan-a cells, we treated the cells with different concentrations of PEDF alone or in combination with H_2_O_2_ for 12 h. The results showed that compared with the control group, treatment with various concentrations of PEDF alone for 12 h did not significantly alter the viability of Melan-a cells; however, the survival rate in the 100 ng/mL PEDF group was significantly higher than that in the 150 and 200 ng/mL groups (*P* < 0.01; Fig. 3A). In the co-treatment with H_2_O_2_ and PEDF, the 50 and 100 ng/mL PEDF groups exhibited a dose-dependent increase in cell survival rate compared to the 150 and 200 ng/mL groups (*P* < 0.01; Fig. 3D), with the highest cell survival rate observed at 100 ng/mL. Compared with the control group, PEDF at 50–100 ng/mL dose-dependently reduced melanin content (*P* < 0.001; Fig. 3B) and inhibited tyrosinase activity (*P* < 0.001; Fig. 3C), with the most significant biological response induced at 100 ng/mL. Higher concentrations (200 and 500 ng/mL) did not further enhance this effect (and even slightly reduced it). A similar trend in melanin content (Fig. 3E) and TYR activity (Fig. 3F) was observed in the co-treatment group with PEDF and H_2_O_2_, suggesting a potential saturation effect. Therefore, 100 ng/mL PEDF was selected for subsequent experiments. Additionally, we observed the morphology of Melan-a cells and found that compared with the control group, Melan-a cells treated with H_2_O_2_ alone exhibited irregular morphology and significantly reduced cell viability (*P* < 0.001), whereas 100 ng/mL PEDF reversed these H_2_O_2_-induced morphological changes and the decrease in cell viability (*P* < 0.01; Fig. 3G, H).

### PEDF alleviated the inhibitory effects of H_2_O_2_ on the migration and proliferation of Melan-a cells

**Fig. 4:**
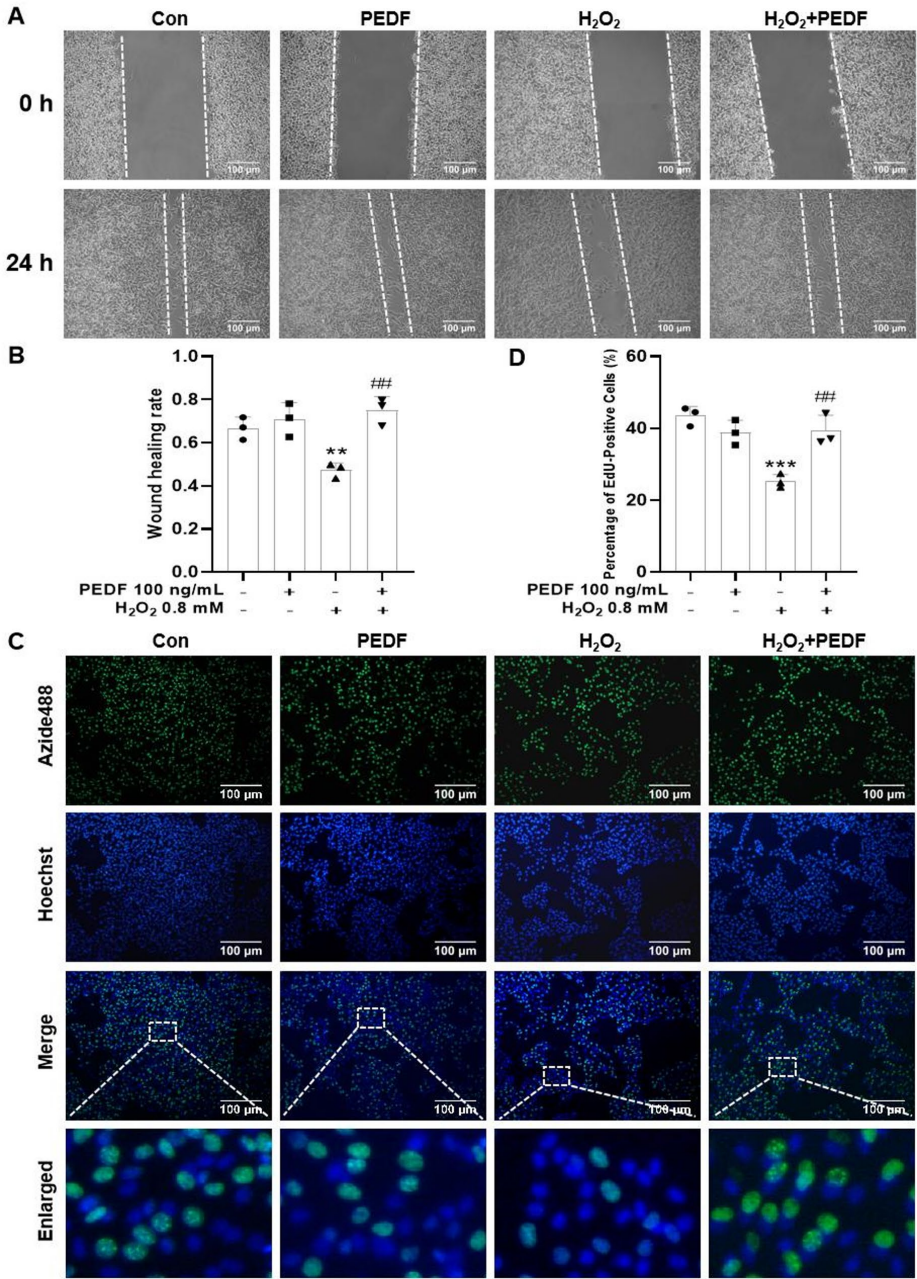
PEDF alleviates the inhibitory effect of H_2_O_2_ on the migration and proliferation of Melan-a cells. **A** Cell migration ability in each group was evaluated using wound-healing assays. Representative images of the wounds at different time points. Scale bar = 100 μm. **B** Quantification of the relative cell migration rate in each group at 24 h. **C** Cells proliferation capacity in each group was determined by the EdU method. Representative images of cell proliferation under different treatments. Scale bar = 100 μm. **D** Quantification of the relative values of cell proliferation rate in each group. The data are presented as the mean ± SD (*n* = 3). ^**^P < 0.01, compared with the control group ; ^#^*P* < 0.05 and ^##^*P* < 0.01, compared with the H_2_O_2_ group

As shown in Fig. 4A and B, compared with the control group, H_2_O_2_ alone treatment group significantly inhibited the migration of Melan-a cells (*P* < 0.01), whereas PEDF significantly reversed H_2_O_2_-induced inhibition of Melan-a cell migration (*P* < 0.01). Similarly, compared with the control group, H_2_O_2_ alone treatment group significantly inhibited the proliferation of Melan-a cells (*P* < 0.01), whereas PEDF significantly alleviated H_2_O_2_-induced inhibition of Melan-a cells proliferation (*P* < 0.01; Fig. 4C, D).

### PEDF alleviated H_2_O_2_-induced cellular oxidative damage

**Fig. 5:**
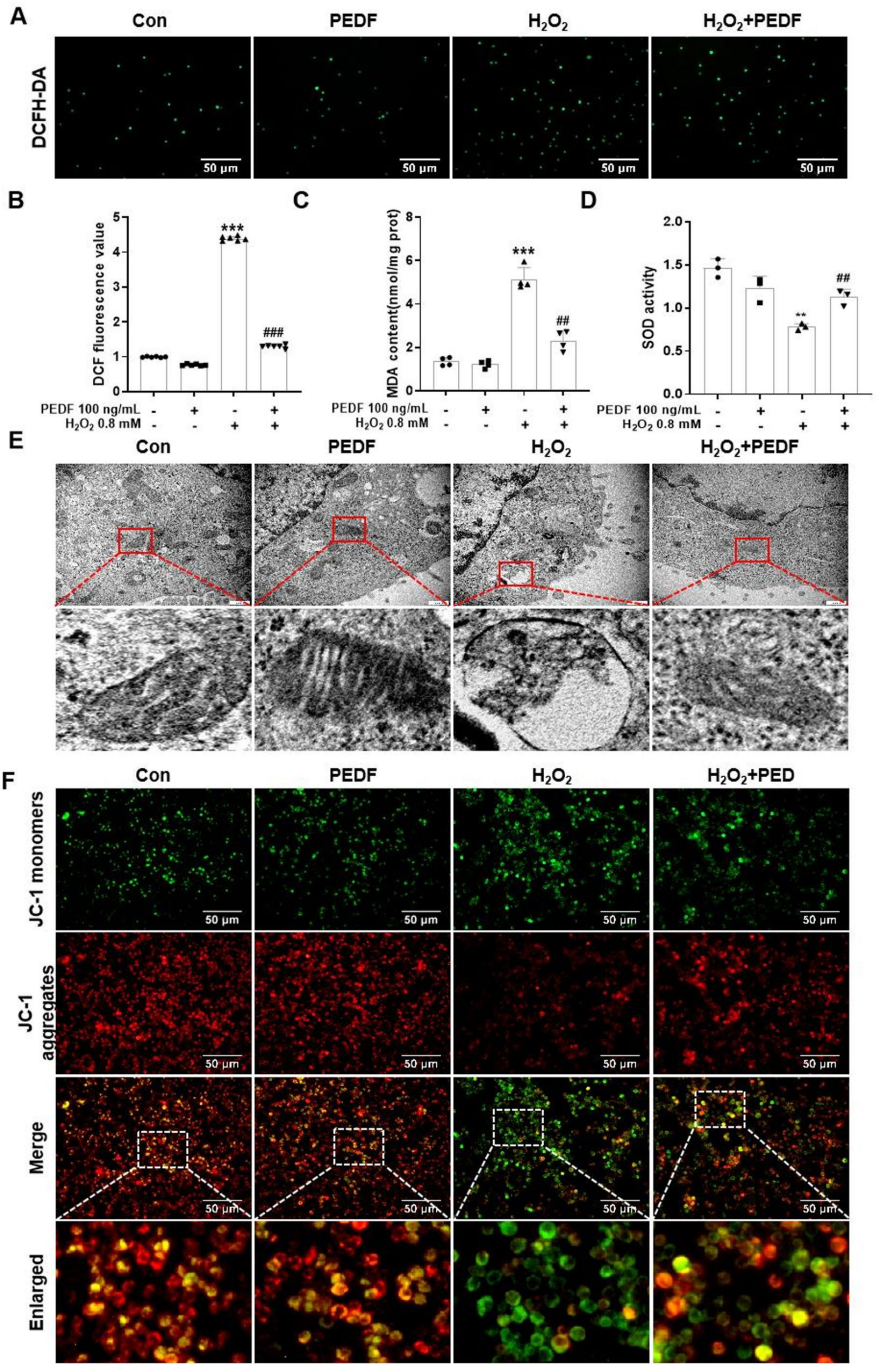
Protective effect of PEDF against H_2_O_2_-induced oxidative stress and mitochondrial dysfunction in Melan-a cells. **A** Detection of intracellular ROS levels in Melan-a cells under different treatments using DCFH-DA fluorescent probe. Scale bar = 50 μm. **B** Quantification of ROS generation in each group of cells. Effects of PEDF on the MDA (**C**) and SOD (**D**) contents in H_2_O_2_-treated Melan-a cells. **E** Representative transmission electron microscope images showing mitochondrial ultrastructural changes in Melan-a cells after treatment with H_2_O_2_ alone or co-treatment with H_2_O_2_ and PEDF for 12 h. Scale bar = 500 μm. **F** Detection of changes in mitochondrial membrane potential in Melan-a cells after different treatments by JC-1 fluorescent probe. Red fluorescence typically represents the fluorescence of JC-1 aggregates under high membrane potential conditions (Ex = 585 nm, Em = 590 nm), indicating normal mitochondrial function; green fluorescence represents the fluorescence of JC-1 monomers under low membrane potential conditions (Ex = 514 nm, Em = 529 nm), suggesting mitochondrial depolarization and functional impairment. Scale bar = 50 μm. The data are presented as the mean ± SD (*n* = 3). ^**^P < 0.01 and ^***^P < 0.001, compared with the control group; ^##^*P* < 0.01 and ^###^*P* < 0.001, compared with the H_2_O_2_ group

To investigate whether PEDF exerts a protective effect against H_2_O_2_-induced oxidative stress, we measured the levels of ROS, SOD, and MDA. Compared with the control group, H_2_O_2_ treatment increased ROS generation in Melan-a cells, whereas PEDF treatment significantly reduced H_2_O_2_-induced ROS production in Melan-a cells (*P* < 0.01, Fig. 5A, B). Compared to the control group, treatment with H_2_O_2_ significantly increased the MDA content in Melan-a cells (*P* < 0.01), whereas the addition of PEDF markedly reduced the H_2_O_2_-induced increase in the MDA content in Melan-a cells (*P* < 0.01, Fig. 5C). Additionally, the H_2_O_2_-treated group presented significantly lower SOD activity than the control group (*P* < 0.01), whereas treatment with 100 ng/mL PEDF markedly increased SOD activity in H_2_O_2_-treated Melan-a cells (*P* < 0.01, Fig. 5D). Mitochondria are the primary sites of ROS production, a process closely linked to cellular energy metabolism, oxidative stress, and signal transduction. Therefore, the ultrastructure of Melan-a cells was examined. The results revealed that in the control group, Melan-a cells presented abundant organelles in the cytoplasm, with mitochondria appearing round or rod shaped, with clearly defined cristae structures, and well-developed rough endoplasmic reticulum. In the PEDF-alone treatment group, mitochondria within the cytoplasm presented an increased electron density, a reduced volume, and well-defined cristae structures. In contrast, compared with control cells, H_2_O_2_-treated Melan-a cells presented swollen or condensed mitochondria in the cytoplasm, with partial dissolution and fragmentation of mitochondrial cristae, along with numerous autophagic-like structures. Notably, cotreatment with PEDF and H_2_O_2_ significantly alleviated H_2_O_2_-induced mitochondrial swelling, restoring clear cristae structures. However, the partial dilation of rough endoplasmic reticulum (RER) cisternae was observed in the cytoplasm (Fig. 5E). To investigate whether PEDF mitigates H_2_O_2_-induced mitochondrial dysfunction, a fluorescence assay was performed using the JC-1 probe. The results demonstrated that after 12 h of treatment, compared with the control, H_2_O_2_ alone significantly increased the proportion of green fluorescence and decreased the red/green fluorescence ratio in the Melan-a cells. In contrast, cotreatment with H_2_O_2_ and PEDF markedly attenuated H_2_O_2_-induced mitochondrial membrane potential (ΔΨm) impairment (Fig. 5F). These results collectively indicate that H_2_O_2_ induced oxidative stress damage in cells and that PEDF alleviated this effect.

### PEDF protects against H_2_O_2_-induced apoptosis in Melan-a cells

**Fig. 6:**
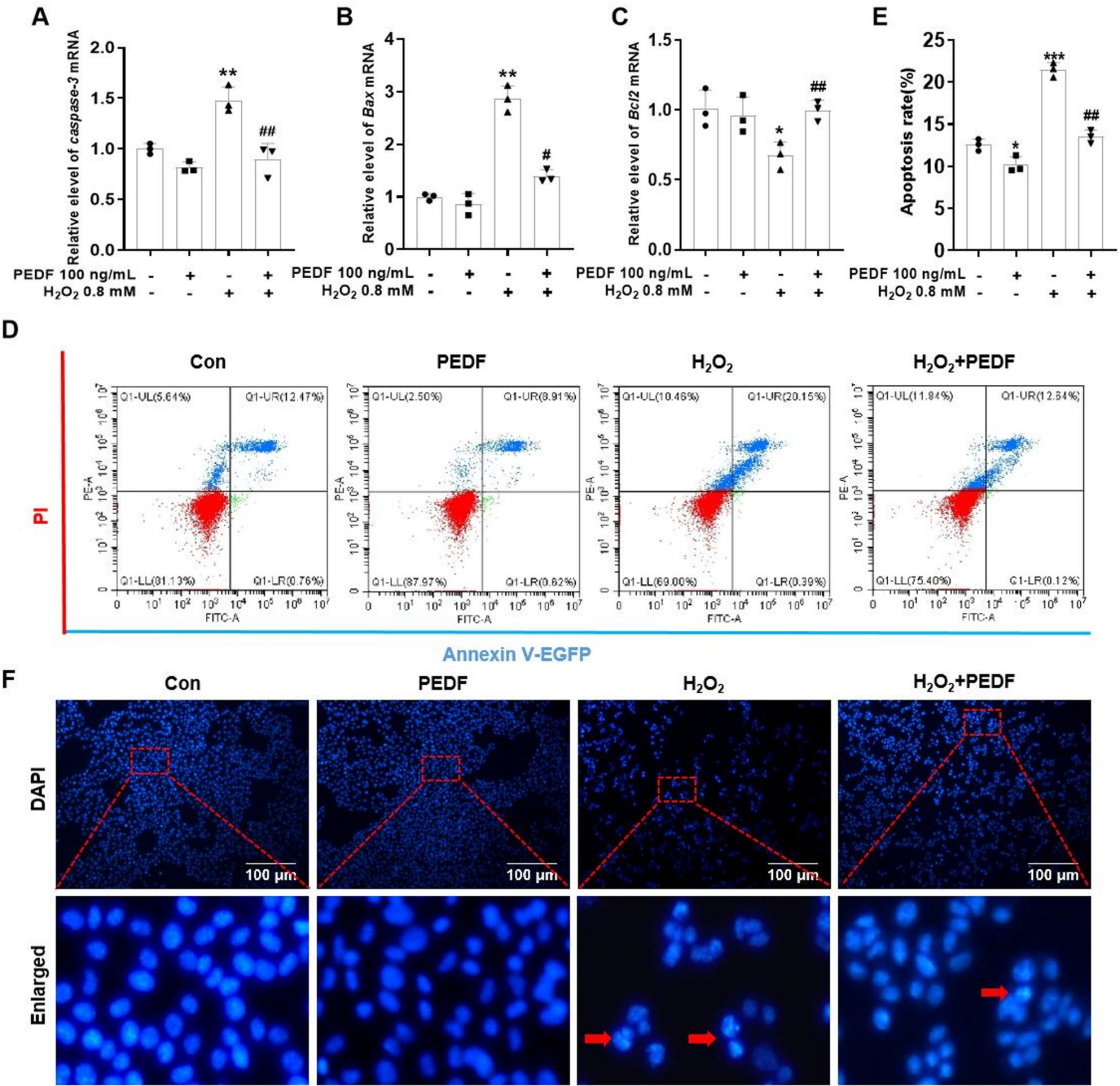
Effects of PEDF on the apoptosis of H_2_O_2_-treated Melan-a cells. The mRNA levels of *Caspase-3* (**A**), *Bax* (**B**), and *Bcl-2* (**C**) in Melan-a cells treatment with H_2_O_2_ alone or co-treatment with H_2_O_2_ and PEDF for 12 h were detected using qRT-PCR. **D** The apoptotic rate was analyzed by flow cytometry after Annexin V-EGFP/PI staining. **E** Quantification of the apoptotic rate in each group of cells. **F** Melan-a cells were fixed with formaldehyde and stained with DAPI to observe changes in cell nuclei. Red arrows indicate apoptotic nuclei. Scale bar = 100 μm. The data are presented as the mean ± SD (*n* = 3). ^*^P < 0.05, ^**^P < 0.01, compared with the control group; ^#^*P* < 0.05 and ^##^*P* < 0.01, compared with the H_2_O_2_ group

To further analyze the protective effect of PEDF against H_2_O_2_-induced apoptosis in Melan-a cells, total RNA was extracted, and qRT-PCR was performed to assess the expression levels of C*aspase-3*, *Bax*, and *Bcl-2*. Compared with the control group, the H_2_O_2_-treated group presented significantly increased mRNA levels of *Caspase-3* and *Bax* and a significant decrease in *Bcl-2* mRNA expression (*P* < 0.01). Compared with H_2_O_2_ treatment alone, PEDF treatment significantly reduced H_2_O_2_-induced *Caspase-3* and *Bax* mRNA expression but markedly increased *Bcl2* mRNA expression in Melan-a cells (*P* < 0.01, Fig. 6A–C). The apoptosis rate of Melan-a cells treated with H_2_O_2_ was analyzed by flow cytometry, as shown in Fig. 6D and E. Compared with the control group, the apoptosis rate of Melan-a cells in the H_2_O_2_-treated group was significantly greater (*P*< 0.01). PEDF treatment significantly inhibited the H_2_O_2_-induced increase in the apoptosis rate of Melan-a cells (*P* < 0.01), and the results were consistent with the qRT-PCR results. To verify the occurrence of apoptosis morphologically, DAPI staining was performed to assess nuclear morphology. The results revealed that Melan-a cells treated with 0.8 mM H_2_O_2_ exhibited irregular morphology and nuclear fragmentation. Notably, treatment with 100 ng of PEDF significantly reversed H_2_O_2_ induced nuclear damage in Melan-a cells (Fig. 6F).

### PEDF decreases the expression levels of Wnt signaling pathway-related genes in H_2_O_2_-treated Melan-a cells

**Fig. 7:**
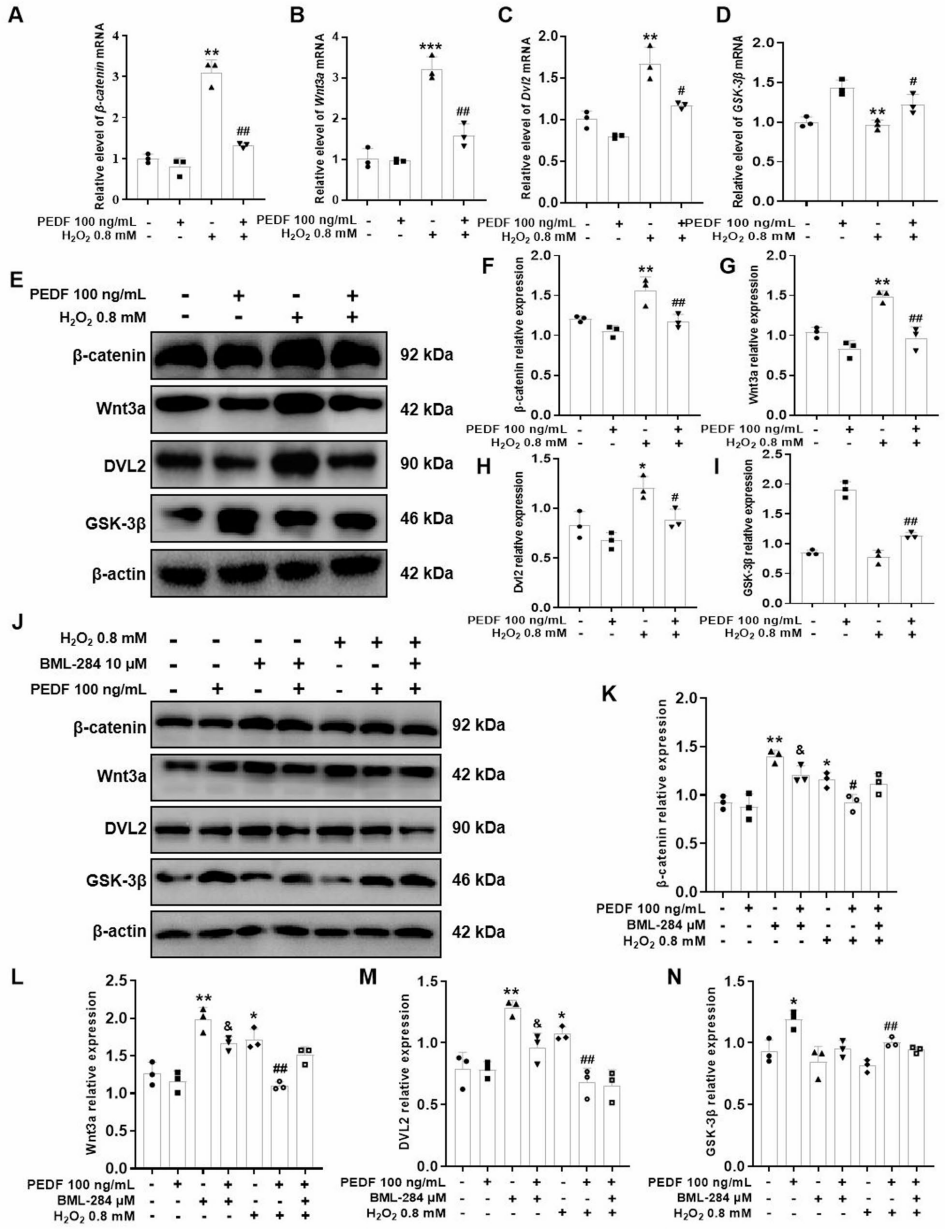
Effects of EPDF on the Wnt/β-catenin signaling pathway in H_2_O_2_-treated Melan-a cells. The mRNA levels of *β-catenin* (**A**), *Wnt3a* (**B**), *DVL2* (**C**), and *GSK-3β* (**D**) in Melan-a cells treatment with H_2_O_2_ alone or co-treatment with H_2_O_2_ and PEDF for 12 h were detected using qRT-PCR. (**E**) The protein levels of β-catenin, Wnt3a, DVL2, and GSK-3β were detected via Western blotting. Representative blots are shown, and β-actin served as a loading control. **F**–**I** Densitometry of representative blots was performed for quantification. **J** Melanocytes were pretreated with 10 µM BML-284 for 2 h, after which BML-284 was removed. The cells were then co-treated with H_2_O_2_ and PEDF for 12 h, and the protein levels of β-catenin, Wnt3a, DVL2, and GSK-3β were measured. Representative blots are shown, and β-actin served as a loading control. **K**–**N** Densitometry of representative blots was performed for quantification. The data are presented as the mean ± SD (*n* = 3). ^*^P < 0.05 and ^**^P < 0.01, compared with the control group; ^#^*P* < 0.05 and ^##^*P* < 0.01, compared with the H_2_O_2_ group; ^&^*P* < 0.05, compared with the BML-284 group

The protein expression levels of Wnt3a, β-catenin, and DVL2 in the 0.8 mM H_2_O_2_-treated group were significantly higher than those in the control group (*P* < 0.01), whereas the level of GSK-3β was significantly lower than that in the control group (*P* < 0.01,); compared with H_2_O_2_ treatment alone, cotreatment with PEDF and H_2_O_2_ significantly reduced the protein expression levels of β-catenin, Wnt3a, and DVL2 (*P* < 0.05; Fig. 7E–I), and the mRNA results were consistent with the protein data (*P* < 0.01; Fig. 7A–D). To verify whether the effect of PEDF on Melan-a cells is dependent on the Wnt/β-catenin signaling pathway, we treated Melan-a cells with the Wnt/β-catenin signaling pathway agonist BML-284. Melan-a cells were treated with BML-284 at concentrations of 0, 5, 10, 20, and 40 µM for 1 h, 2 h, or 3 h, respectively. The results showed that BML-284 exerted an inhibitory effect on cell viability in both a time-dependent and dose-dependent manner (Fig. S1A). Western blotting analysis was also performed to detect the effect of BML-284 on β-catenin protein levels. The results showed that BML-284 significantly increased β-catenin protein levels in a dose-dependent manner (Fig. S1B). CCK-8 assay and Western blotting demonstrated that 10 µM BML-284 significantly increased the expression level of β-catenin while not affecting cell viability. Therefore, the dose of 10 µM was selected for subsequent experiments. After treatment with BML-284, compared with the control group, PEDF still significantly reversed the increases in the protein levels of β-catenin, Wnt3a, and DVL2 (*P* < 0.01), and the expression of GSK-3β was markedly elevated (*P* < 0.05; Fig. 7J–N). These results suggest that PEDF can inhibit the activation of the Wnt/β-catenin signaling pathway.

### PEDF reduces H_2_O_2_-induced apoptosis in Melan-a cells by inhibiting the Wnt/β-catenin signaling pathway

**Fig. 8:**
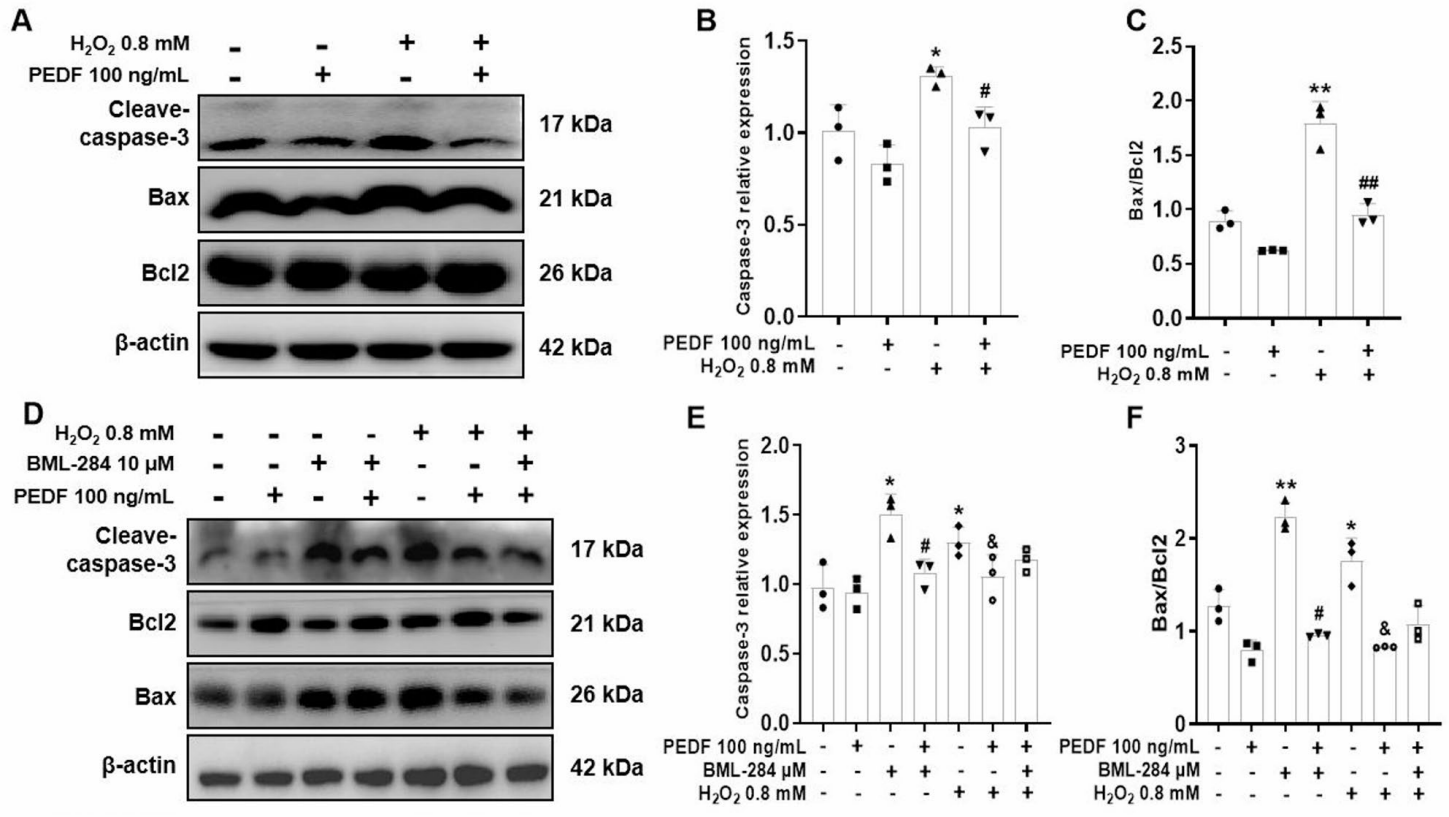
Effects of PEDF on the protein levels of Caspase-3, Bax, and Bcl2 in H_2_O_2_-treated melan-a cells. **A** The protein levels of apoptosis-related markers (Caspase-3, Bax, and Bcl2) in Melan-a cells treatment with H_2_O_2_ alone or co-treatment with H_2_O_2_ and PEDF for 12 h were detected via Western blotting. Representative blots are shown, and β-actin served as a loading control. **B**, **C** Densitometry of representative blots was performed for quantification. **D** Melanocytes were pretreated with 10 µM BML-284 for 2 h, after which BML-284 was removed. The cells were then co-treated with H_2_O_2_ and PEDF for 12 h, and the protein levels of Caspase-3, Bax, and Bcl-2 were measured. Representative blots are shown, and β-actin served as a loading control. **E**, **F** Densitometry of representative blots was performed for quantification. The data are presented as the mean ± SD (*n* = 3). ^*^P < 0.05 and ^**^P < 0.01, compared with the control group; ^#^*P* < 0.05, compared with the BML-284 group; ^&^*P* < 0.05 and ^&&^*P* < 0.01, compared with the H_2_O_2_ group

To determine whether H_2_O_2_ induces apoptosis through the Wnt/β-catenin signaling pathway, we assessed the expression of apoptosis-related genes and proteins. Compared with the control group, H_2_O_2_ significantly increased the expression of Caspase-3 and Bax and decreased the expression of Bcl-2. However, these effects were reversed by PEDF treatment, which markedly reduced H_2_O_2_ -induced expression of Caspase-3 and Bax expression and increased Bcl-2 expression (Fig. 8A–C), indicating that PEDF can protect Melan-a cells from H_2_O_2_-induced apoptosis. After pretreatment with BML-284, PEDF still significantly decreased H_2_O_2_ -induced the protein expression of Bax and Caspase-3 (*P* < 0.01) but markedly increased the expression of Bcl-2 (*P* < 0.05; Fig. 8D–F). These results indicate that PEDF inhibits apoptosis by suppressing the activation of the Wnt/β-catenin signaling pathway.

### PEDF modulates the expression of melanogenesis markers in H_2_O_2_-treated Melan-a cells

**Fig. 9:**
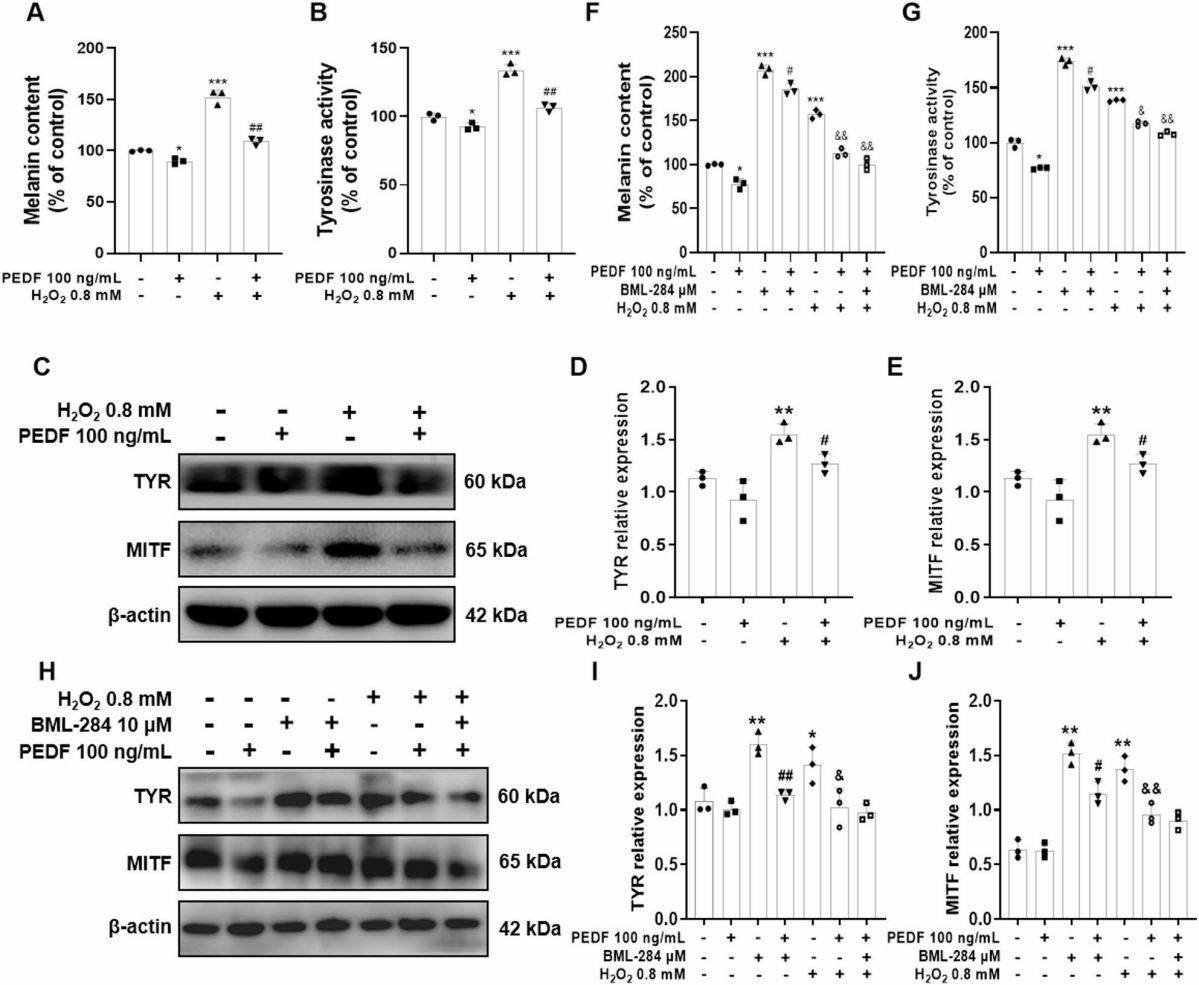
Effects of PEDF on H_2_O_2_-induced melanin production in melanin-a cells. **A** The melanin content in Melan-a cells treated with H_2_O_2_ alone or co-treated with H_2_O_2_ and PEDF for 12 h was detected by alkaline lysis method. **B** The TYR activity in Melan-a cells treated with H_2_O_2_ alone or co-treated with H_2_O_2_ and PEDF for 12 h was detected by spectrophotometry (L-DOPA oxidation method). **C** The protein levels of TYR and MITF were detected via Western blotting. Representative blots are shown, and β-actin served as a loading control. **D, E** Densitometry of representative blots was performed for quantification. **F** Melan-a cells were pretreated with 10 µM BML-284 for 2 h, after which BML-284 was removed. The cells were then co-treated with H_2_O_2_ and PEDF for 12 h, and the melanin content in Melan-a cells was detected by alkaline lysis method. **G** The TYR activity in Melan-a cells was detected by spectrophotometry. **H** The protein levels of TYR and MITF were detected via Western blotting. Representative blots are shown, and β-actin served as a loading control. **I, J** Densitometry of representative blots was performed for quantification. The data are presented as the mean ± SD (*n* = 3). ^*^P < 0.05 and ^**^P < 0.01, compared with the control group; ^#^*P *< 0.05 and ^##^*P* < 0.01, compared with the BML-284 group; ^&^*P* < 0.05 and ^&&^*P* < 0.01, compared with the H_2_O_2_ group

To confirm the inhibitory effect of PEDF on Wnt/β-catenin signaling pathway and its impact on melanocyte function, we examined melanin content, tyrosinase (TYR) activity, and the expression of melanogenesis markers. Compared with the control group, the H_2_O_2_-treated group exhibited a 1.4-fold increase in melanin levels and a 1.25-fold increase in TYR activity, whereas PEDF treatment reversed these effects (1.09-fold reduction in melanin and 1.35-fold reduction in TYR activity compared to the H_2_O_2_ alone group, *P* < 0.001) (Fig. 9A, B). Consistently, compared with the control group, 0.8 mM H_2_O_2_ activated melanin synthesis, significantly increasing the protein expression levels of TYR and MITF, whereas PEDF treatment suppressed the H_2_O_2_-induced generation of melanin markers (Fig. 9C–E). Similar to H_2_O_2_ treatment, pretreatment with the Wnt agonist BML-284 significantly increased melanin content, TYR activity, and melanogenic marker expression in Melan-a cells compared to the control group; however, PEDF significantly reduced BML-284-induced melanin content, TYR activity, and melanogenic marker expression in Melan-a cells (*P* < 0.01; Fig. 9F–J), suggesting that PEDF may attenuate melanogenesis by inhibiting the Wnt/β-catenin signaling pathway.

## Discussion

Studies have shown that ROS are continuously generated during normal physiological metabolism and participate in regulating various physiological processes (e.g., cellular metabolism). Cells maintain low homeostatic levels of ROS through antioxidant systems, and impairment of these systems leads to excessive ROS accumulation, which in turn triggers pathological processes such as vitiligo, melanoma, and neurological disorders [38, 39]. As a key member of ROS, H_2_O_2_ contributes to the maintenance of redox homeostasis by regulating metabolism and stress responses [40]. However, its excessive presence reacts with metal ions to generate hydroxyl radicals, resulting in oxidative damage [41]. Moreover, under physiological conditions, H_2_O_2_ exerts regulatory effects on various physiological processes including cell proliferation, differentiation, and migration [42]. Previous studies have shown that low concentrations of H_2_O_2_ can reduce the expression levels of the melanogenesis-related genes MITF and TYR, whereas treatment with 1 mM H_2_O_2_ upregulates the expression of these genes. This increase triggers elevated intracellular ATP and cAMP levels, ultimately inducing both apoptosis and melanogenesis [43]. This study found that the effect of H_2_O_2_ on Melan-a cells exhibits concentration dependence: low concentrations of H_2_O_2_ (0.4 mM) decreased the expression of MITF and TYR, whereas higher concentrations (≥ 0.6 mM) upregulated their expression. Furthermore, treatment with increasing H_2_O_2_ concentrations led to a reduction in the expression of Wnt pathway-related proteins and an increase in the expression of apoptosis-associated proteins. Specifically, treatment with 0.8 mM H_2_O_2_ significantly decreased cell viability and increased cell mortality rate, while inducing elevated levels of ROS and MDA, inhibited SOD activity, and increased apoptosis. These results confirm that this concentration can effectively establish an oxidative stress model, providing an experimental basis for subsequent studies on the antioxidant therapeutic effects of PEDF.

PEDF, a pleiotropic protein belonging to the serine protease inhibitor (serpin) superfamily, has demonstrated antiangiogenic, anti-inflammatory, and antioxidant effects in both cell culture and animal models [44, 45]. Numerous studies have investigated the antioxidant properties of PEDF. For example, Nemerovsky L et al. demonstrated that PEDF protects oocytes by modulating oxidative stress [46]. Our study revealed that after H_2_O_2_ treatment, Melan-a cells presented morphological alterations, decreased cell viability, increased mortality rates, and suppressed proliferation and migration. However, the addition of PEDF reversed the H_2_O_2_-induced morphological changes in Melan-a cells and increased cell viability, decreased the mortality rate, and alleviated the suppression of cell proliferation and migration. Mitochondria are the primary source of cellular ROS, where superoxide is rapidly converted to H_2_O_2_ - a key mediator of redox signaling and oxidative stress [47]. While mitochondria efficiently metabolize H_2_O_2_ under physiological conditions, pathological states (e.g., cancer) disrupt this balance, leading to ROS accumulation and mitochondrial dysfunction [48]. Excessive ROS damage mitochondrial DNA, proteins, and membrane integrity, impairing ATP production and membrane potential (ΔΨm) [49]. In ROT-induced senescent RPE cells, PEDF protects cristae and mitochondrial membrane integrity, increases ATP levels and ΔΨm, and reduces ROS levels [45]. MDA is generated by free radical-mediated lipid peroxidation, and SOD is the only enzyme that specifically interacts with superoxide, helping to maintain ROS levels in cells [41, 50]. Ma S et al. reported that PEDF pretreatment reduces ox-LDL-induced ROS and MDA production and increases SOD activity, whereas PEDF-siRNA significantly enhances ox-LDL-induced oxidative stress [51]. Our results show that H_2_O_2_ induces mitochondrial swelling, cristae fragmentation, and ROS/MDA accumulation in Melan-a cells, while suppressing SOD activity. The addition of PEDF significantly reduced the H_2_O_2_-induced generation of ROS and MDA, increased SOD activity, and ameliorated the mitochondrial swelling caused by H_2_O_2_. The mitochondrial cristae structure became distinct, and the decrease in the mitochondrial membrane potential induced by H_2_O_2_ was alleviated, indicating that PEDF can mitigate H_2_O_2_-induced mitochondrial damage in Melan-a cells.

Many studies have reported that excessive intracellular ROS generation induces mitochondrial damage and oxidative stress. Impaired mitochondria activate and amplify apoptotic signaling through multiple pathways, ultimately leading to cell death via uncontrolled oxidative stress-driven mitochondrial apoptosis [52–54]. Studies have revealed that the Bcl-2 family (arbiters of the mitochondrial apoptosis pathway) can process and release cytochrome C (Cyt C) through proapoptotic members such as Bax, thereby promoting the aggregation of procaspase-9 in the cytoplasm and the activation of downstream Caspase-3, which ultimately induces apoptosis [55, 56]. Many studies have demonstrated that H_2_O_2_ can increase intracellular ROS levels and induce apoptosis [57–59]. Additional studies have demonstrated that PEDF inhibits apoptosis through multiple signaling pathways [60–62]. Notably, in this study, H_2_O_2_ significantly increased the mRNA levels of Bax and Caspase-3 but decreased those of Bcl-2 in Melan-a cells. However, these H_2_O_2_-induced alterations in apoptosis-related mRNA levels were markedly reversed after PEDF treatment. Moreover, flow cytometry and DAPI nuclear staining results demonstrated that PEDF significantly alleviated H_2_O_2_-induced apoptosis in Melan-a cells. These findings suggest that H_2_O_2_ can induce apoptosis in Melan-a cells, whereas PEDF inhibits H_2_O_2_-induced Melan-a cell apoptosis.

The Wnt/β-catenin signaling pathway plays a critical role in oxidative stress-induced cell apoptosis. Previous studies have shown that Wnt3a activates the Wnt/β-catenin signaling pathway by increasing phosphorylated LRP6 (pLRP6) levels in ARPE19 cells (a human retinal pigment epithelial cell line), whereas PEDF, an endogenous inhibitor of this pathway, suppresses β-catenin through the inhibition of Wnt3a in a dose-dependent manner [63]. A growing body of research has demonstrated that the Wnt/β-catenin pathway is involved in oxidative stress-induced apoptosis. Qiu L et al. reported that H_2_O_2_ activates the Wnt/β-catenin pathway and induces oxidative damage in H9c2 cardiomyocytes [64]. Ma S et al. reported that PEDF ameliorates endothelial injury by inhibiting the Wnt/β-catenin signaling pathway, reducing the oxidative stress response, and thereby exerting antiapoptotic effects [51]. This study further confirmed that both H_2_O_2_ and the Wnt agonist BML-284 significantly increased the protein and mRNA levels of *Wnt3a*, *β-catenin*, and *DVL2* in Melan-a cells, while decreased the level of *GSK-3β*, accompanied by the activation of *Caspase-3* and *Bax*. PEDF treatment significantly reversed these effects, suggesting that it alleviates H_2_O_2_-induced oxidative stress apoptosis by inhibiting the Wnt/β-catenin signaling pathway. Notably, that after the cells were treated with PEDF, H_2_O_2_ and BML-284 together, the expression levels of the Wnt3a, β-catenin and DVL2 proteins were lower than those in the groups in which PEDF was cotreated with H_2_O_2_ and BML-284, respectively, whereas the expression level of GSK-3β was higher than that in the groups in which PEDF was cotreated with H_2_O_2_ and BML-284, respectively. This result requires further research and verification.

Melanocytes, which originate from the neural crest (a neuroectodermal derivative), are identified by markers such as DCT, TRP1, TYR, and MITF, and their pigment synthesis is regulated by multiple pathways [65, 66]. Hu et al. reported that UVB irradiation induces mitochondrial damage in melanocytes, triggering excessive ROS production, which leads to the upregulation of melanogenesis-related gene expression [67]. We also observed consistent results: low concentrations (0.4 mM) of H_2_O_2_ decreased the expression of MITF and TYR, whereas higher concentrations (≥ 0.6 mM) increased their expression. Previous studies have demonstrated that the Wnt/β-catenin signaling pathway is a critical pathway for melanin synthesis in melanocytes. Wnt1 or β-catenin promotes the proliferation and differentiation of neural crest cells into melanocytes in vivo, whereas reducing the expression of Wnt1, Wnt3a, and β-catenin decreases melanin production [68]. Wnt3a treatment significantly enhances TYR activity and increases melanin synthesis in mouse melanocytes, whereas the inhibition of the Wnt/β-catenin signaling pathway markedly suppresses melanin production [69]. We observed that both H_2_O_2_ and BML-284 activated the Wnt/β-catenin signaling pathway and upregulated the expression of melanogenesis-related genes, whereas PEDF inhibited the Wnt/β-catenin signaling pathway, thereby reducing the expression of MITF and TYR and further suppressing melanin synthesis. These results suggest that PEDF inhibits melanogenesis, likely through the suppression of the Wnt/β-catenin signaling pathway.

## Conclusion

These findings collectively suggest that the Wnt/β-catenin signaling pathway may mediate H_2_O_2_-induced oxidative damage and melanin production in Melan-a cells through oxidative stress, whereas PEDF enhances the antioxidant capacity by inhibiting the Wnt/β-catenin signaling pathway, thereby reducing both apoptosis and melanin synthesis.

## Supplementary Information

Below is the link to the electronic supplementary material.

Supplementary Material 1

Supplementary Material 2

## Data Availability

The data that support the findings of this study are available from the corresponding author upon reasonable request.

## Abbreviations

PEDF: Pigment epithelium derived factor
H_2_O_2_: Hydrogen peroxide
LDH: lactate dehydrogenase
TEM: Transmission electron microscopy
ROS: Reactive oxygen species
RNS: Reactive nitrogen species
SOD: Superoxide Dismutase
MDA: Malondialdehyde
MITF: Microphthalmia-associated transcription factor
TYR: Tyrosinase
TRP-1: Tyrosinase-related protein 1
DCT: Dopachrome tautomerase
GSK-3β: Glycogen synthase kinase-3β
