# Supplementary material for: Influence of pigment epithelium-derived factors on H2O2-induced oxidative damage and melanin synthesis in Melan-a cells
Source: Biol Res. 2025 Dec 29;58:78. doi: 10.1186/s40659-025-00657-8 (PMC12751373; doi:10.1186/s40659-025-00657-8)
Supplement: Supplementary file 1 — Supplementary Material 1 [file 40659_2025_657_MOESM1_ESM.docx]

**Supplement**

**Figures and table included**

Influence of pigment epithelium-derived factors on H_2_O_2_-induced oxidative damage and melanin synthesis in Melan-a cells

(Manuscript ID BRES-D-25-00570)

Fig.S1


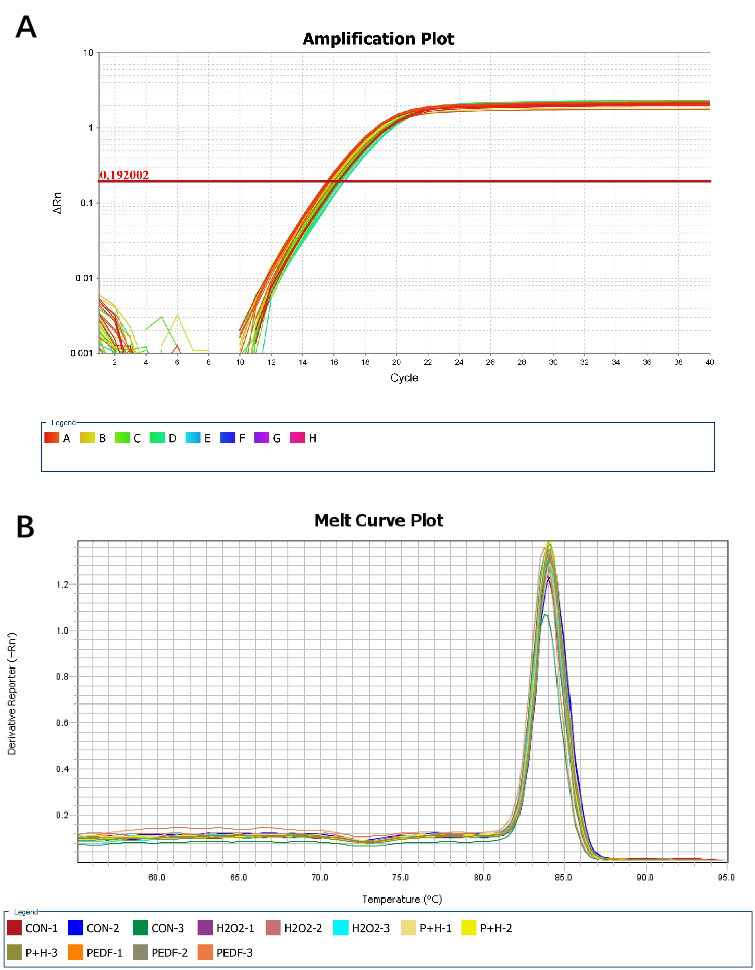


**Fig. S1 Amplification curves and melting curves of the endogenous reference gene β-actin.**

Melan-a cells were treated with H₂O₂ alone or co-treated with H₂O₂ and PEDF for 12 h. Quantitative real-time PCR was performed using SYBR® Green dye on a QuantStudio 5 instrument to validate the amplification of the endogenous reference gene β-actin. (A) β-actin Amplification curves exhibited stable baselines, clear inflection points, and absence of primer dimers, indicating robust and specific amplification kinetics. (B) β-actin Melting curves displayed a single sharp peak, confirming the specificity of the amplified product with no non-specific byproducts.

Fig.S2


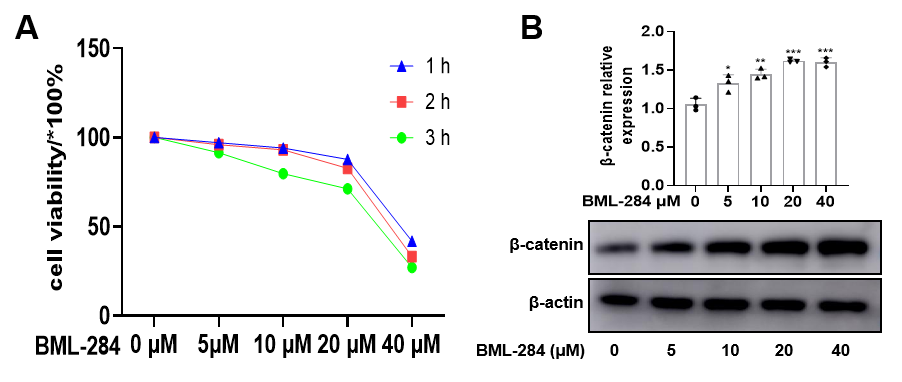


**Fig. S2 The effect of PEDF on Melan-a cell viability and β-catenin protein expression levels.**

(A) Melanocytes were treated with BML-284 at concentrations of 0, 5, 10, 20, and 40 μM for 1 h, 2 h, or 3 h, respectively, and the effect of BML-284 on cell viability was evaluated using the CCK-8 assay. (B) Melanocytes were treated with BML-284 at concentrations of 0, 5, 10, 20, and 40 μM for 2 h, after which the protein level of β-catenin was measured. A representative electrophoresis image is shown, with β-actin used as the loading control. The data are presented as the mean ± SD (n=3). ^*^*P* < 0.05, ^**^*P* < 0.01 and ^***^*P* < 0.001 compared with the control group.
